# IsoPrimer: a pipeline for designing isoform-aware primer pairs for comprehensive gene expression quantification

**DOI:** 10.1093/bioadv/vbaf171

**Published:** 2025-07-15

**Authors:** Ermes Filomena, Ernesto Picardi, Graziano Pesole, Anna Maria D’Erchia

**Affiliations:** Department of Biosciences, Biotechnology and Environment, University of Bari Aldo Moro, Bari 70125, Italy; Department of Biosciences, Biotechnology and Environment, University of Bari Aldo Moro, Bari 70125, Italy; Institute of Biomembranes, Bioenergetics and Molecular Biotechnologies, National Research Council, Bari 70126, Italy; Department of Biosciences, Biotechnology and Environment, University of Bari Aldo Moro, Bari 70125, Italy; Institute of Biomembranes, Bioenergetics and Molecular Biotechnologies, National Research Council, Bari 70126, Italy; Department of Biosciences, Biotechnology and Environment, University of Bari Aldo Moro, Bari 70125, Italy; Institute of Biomembranes, Bioenergetics and Molecular Biotechnologies, National Research Council, Bari 70126, Italy

## Abstract

**Motivation:**

Eukaryotic genes can perform different functions by generating multiple transcripts through the alternative splicing mechanism. The accurate quantification of gene expression in specific conditions is important for functional assessment and requires an accurate PCR primer pair design to target all expressed alternative transcripts, a complex and prone-to-error task if performed manually.

**Results:**

To efficiently address this task, we developed a pipeline, called IsoPrimer, to design PCR primer pairs targeting the specific set of expressed splicing variants of the genes of interest, to be used in quantitative PCR, e.g. in RNA-seq validation experiments. IsoPrimer, according to the level of expression of the splicing variants derived from an RNA-seq dataset, can: (i) identify the most expressed gene isoforms; (ii) design primer pairs overlapping exon-exon junctions common to the expressed variants; (iii) verify the specificity of the designed primer pairs.

**Availability and implementation:**

IsoPrimer is available for download from https://github.com/BioinfoUNIBA/IsoPrimer

## 1 Introduction

The discontinuous nature of eukaryotic genes requires the splicing process to produce functional transcripts. Although appearing as a superfluous complication of the exploitation of genetic information, splicing significantly expands the gene’s functional potential, as different RNA molecules, called isoforms, can be generated from a single precursor through the alternative splicing (AS) mechanism. Thus, to investigate the functional activity of eukaryotic genes, a comprehensive assessment of the expression level of the different gene isoforms is mandatory.

The deep sequencing-based analysis of the transcriptome (RNA-seq) is a valuable and widely used high-throughput assay to investigate the gene expression profile of tissues (multiple cellular types, bulk RNA-seq) or single cells (scRNA-seq), qualitatively and quantitatively. The digital nature of RNA-seq-based experimental designs leads to the parallel measurement of the expression of numerous genes through the mapping of sequencing reads on the reference genome and the subsequent transcript quantification through suitable software, as FeatureCounts (Liao, Smyth and Shi 2014). Usually, an RNA-seq analysis is followed by an in vitro validation, carried out by quantitative PCR-based methods, such as Reverse Transcription quantitative PCR (RT-qPCR) or digital droplet PCR (ddPCR). In specific cellular conditions, only a subset of the transcripts of a gene, which can be generated by alternative splicing, is expressed. Accordingly, the design of PCR primers to validate gene expression measured through RNA-seq, targeting all the expressed transcripts of a gene, is not trivial, especially in the case of complex splicing patterns.

In recent years, several tools have been developed to facilitate the PCR primer pairs design, as comprehensively reviewed by Guo *et al.* (2021), which classified different PCR primer designing tools based on their functionalities. Tools such as AutoPrime (Wrobel *et al.* 2004), QuantPrime (Arvidsson *et al.* 2008), and the widely used, high-performance Primer-BLAST (Ye *et al.* 2012) offer user-friendly interfaces for general PCR primer design. Other tools, as PRIMEGENS-v2 (Srivastava *et al.* 2011), RASE (Brosseau *et al.* 2010), and PrimerSeq (Tokheim *et al.* 2014) belong to the Splicing Variant category as they facilitate the PCR primer pairs design for the detection of AS variants. Notably, the PRIMEGENS-v2 software can design homolog-specific primers for amplifying each alternatively spliced variant of a gene, excluding transcripts of its paralogs. The RASE (real-time PCR annotation of splicing events) pipeline can automatically design specific primer pairs for 81% of all human alternative splicing events present in the NCBI build 36 database, but without considering the expression level of splice variants in the tissues. The PrimerSeq tool incorporated RNA-seq data in the design and visualization of RT-PCR primers for the analysis of specific alternative splicing variants. It leverages information from RNA-seq alignment outputs, e.g. BAM/SAM files, to provide a graphical representation of the expected RT-PCR results.

In this context, there are no available tools able to guide researchers in designing PCR primer pairs to carry out an accurate quantification of gene expression, e.g. to validate RNA-seq analysis, taking into account all the expressed transcripts of a gene. In fact, current tools cannot prioritize designed primers based on the estimated abundance of alternative transcripts and/or they can be used only as a web service.

**Figure 1.**
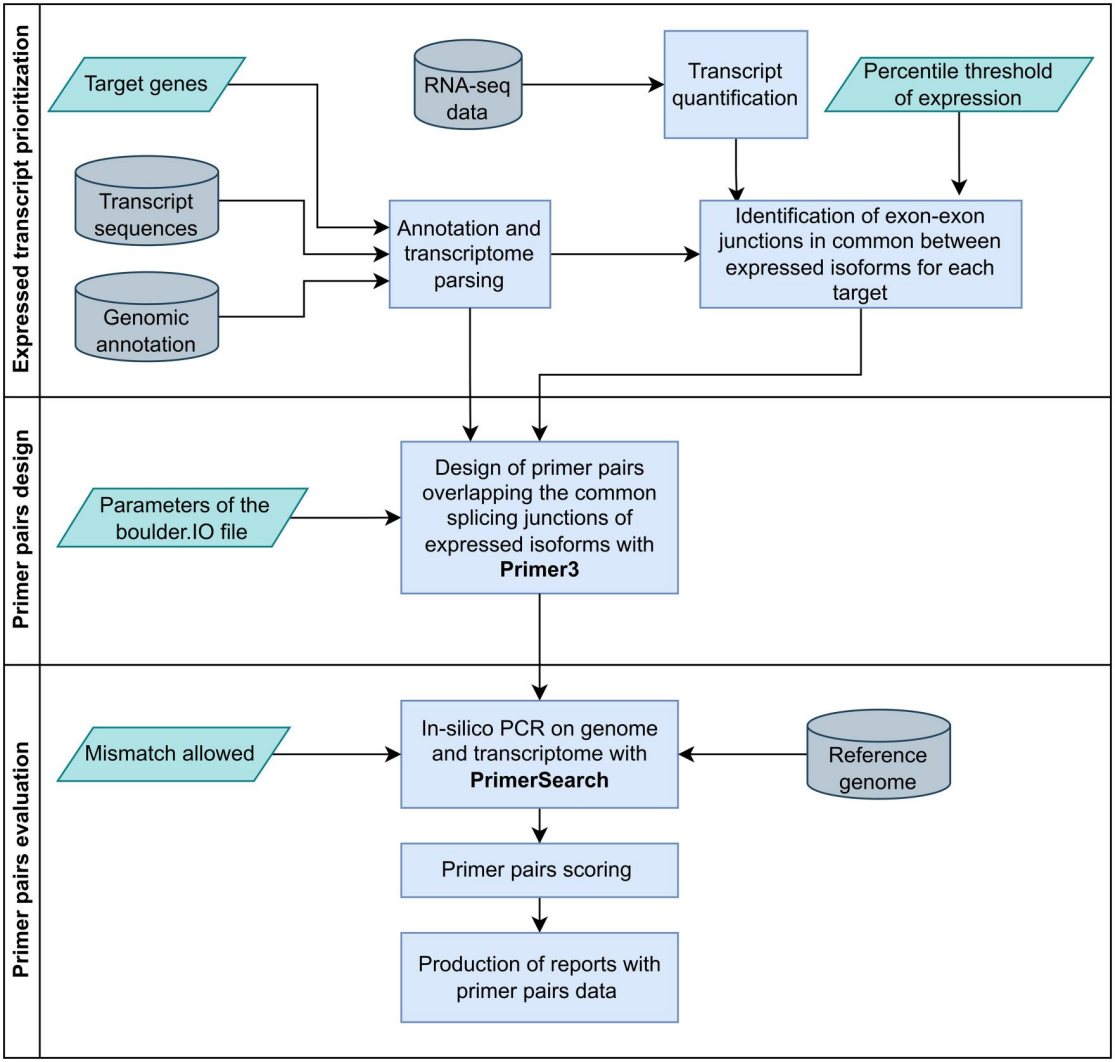
Flowchart of the IsoPrimer pipeline. Overview of the IsoPrimer pipeline workflow, showing the sequence of steps for designing primer pairs specific to the expressed variants of a gene. Input data is represented as cylinders, processes are described in rectangular boxes, while user-defined parameters such as the expression threshold, Primer3 parameters and maximum mismatch allowance for in-silico PCRs are in oblique boxes. The pipeline leverages the quantification of transcripts (optionally performed with the built-in Kallisto tool) and the Primer3 and EMBOSS PrimerSearch tools, respectively to: (i) identify the most expressed isoforms per gene in RNA-seq samples; (ii) design and consider all primer pairs overlapping exon-exon junctions common to the expressed variants; (iii) verify the specificity of the primer pairs designed.

Given the extraordinary complexity and specificity of gene expression across different cellular contexts and conditions, primer design is significantly more effective and accurate when informed by RNA-seq data, especially considering the previously unknown exon–exon junctions that are continuously being discovered. In fact, many genes can produce a large number of isoforms, with few or even no exon–exon junctions shared across all transcripts. A representative example is MEG8, a non-coding RNA gene composed of 37 exons and 65 annotated isoforms, as reported in GTEx portal (Fig. 1, available as supplementary data at *Bioinformatics Advances* online). To tackle this issue, primer pairs can be designed specifically for the exons and exon–exon junctions shared by a set of expressed splicing variants of a gene, which indeed require RNA-seq data. This is a critical aspect to consider when designing primer pairs for qPCR experiments aimed at assessing gene expression. Since the above considerations, we developed IsoPrimer, an automated pipeline that, taking into account the estimated level of expression of the splicing variants of a gene, as derived from RNA-seq data, performs a prioritization of the designed primer pairs. Isoprimer can assist researchers in automating a task that would otherwise be complex and error-prone if performed manually.

## 2 Implementation and results

IsoPrimer is designed to be executed on machines running GNU+Linux, it is freely available for download under the GNU v.3 public license from the following link (https://github.com/BioinfoUNIBA/IsoPrimer), and it may be inspected and modified by the user as required. The IsoPrimer workflow is summarized in Fig. 1.

### 2.1 Software requirements

The IsoPrimer pipeline is written in R (version 3.6.1). The child scripts dedicated to handling other programs included in the pipeline, i.e. Kallisto (version 0.48) (Bray *et al.* 2016), Primer3 (libprimer3 release 2.6.1) (Untergasser *et al.* 2012) and EMBOSS PrimerSearch (version 6.6.0.0) (Rice *et al.* 2000), are written in Bash.

Kallisto, Primer3 and EMBOSS PrimerSearch may be downloaded and compiled according to their respective documentation, but they are available as Conda packages, as well. R and Bash need to be installed on the system for the IsoPrimer pipeline to function as expected. The following R packages and their dependencies are also required:

- dplyr, version 0.8.3;
- BiocManager, version 1.30.10;
- Biostrings, version 2.54.0;
- doParallel, version 1.0.16;
- openxlsx, version 4.1.5;
- stringr, version 1.4.0;
- msa, version 1.18.0.

At the time of its first execution, IsoPrimer will attempt to load or automatically install the missing package(s) if necessary.

### 2.2 IsoPrimer workflow

Once the pipeline options are defined by the user within the “launcher.sh” script, the pipeline can be launched to perform the following analytical steps:

1. Choice of the expressed isoforms for primer design. User supplies the pipeline a transcript quantification table, formatted as the “kalcounts.tsv” file, obtained with a quantification tool external to the pipeline or manually populated with arbitrary expression values to prioritize specific sets of isoforms. Alternatively, if no table is provided, it can be generated by the default transcript quantifier, Kallisto. IsoPrimer then parses the table to identify and rank the most highly expressed transcript variants and designs multiplex primer pairs accordingly. For example, to specifically target the murine Odc gene’s Odc1‑207 variant (Ensembl ID: ENSMUST00000222250), a user can provide a kalcounts.tsv file structured as in Table 1, available as supplementary data at *Bioinformatics Advances* online.
2. The genomic annotation (.gtf file) and the reference transcriptome (transcript sequences in FASTA format) are processed to gather all information required to design primers for each gene of interest, the most relevant of which are: (i) gene ID; (ii) IDs, sequences, strand and length of its transcripts; (iii) position of splicing junctions in the transcripts; (iv) if using the GENCODE or ENSEMBL annotation, transcript support level (TSL) of each isoform; only transcripts with TSL ≤ 3 are considered as templates for the design of primer pairs.
3. A 10 nt long sequence spanning each splice junction (5 nt upstream and downstream of the site) is searched within the transcripts of the target genes. The matches are used to rank the splicing junctions based on their prevalence across isoforms.
4. For each target gene, for each splicing variant, Primer3 is launched recursively to design 5 primer pairs for each splicing junction, so that one primer of the pair overlaps the junction. Primer3 is instructed with the parameters which are specified in the Boulder-IO file “rock.bakup.” The default options listed in the file (except for the template sequence, the position of the exon-exon junction in the transcript sequence which are filled by the pipeline) may be modified by the user according to the Primer3 manual (see https://primer3.org/manual.html), e.g.: primer length (20–25 bases); G/C content (30%–60%); melting temperature (Tm) (55–75°C); amplicon length (100–300 bp); maximum length of poly-N stretches (4 bases). By default, Primer3 designs primers, avoiding self and pair complementarity between primers. All unique primer pairs designed by Primer3 are gathered in a table.
5. To assess the specificity of the primer pairs designed, an in-silico PCR is performed with each pair, by using the EMBOSS PrimerSearch tool and the transcriptome sequences reported in the FASTA file provided by the user, as a template. By default, for the in-silico PCR, a 20% mismatch with the template is allowed for each primer, but users may define the parameter by editing the dedicated line of the “launcher.sh” script.
6. PrimerSearch results are then processed, prioritizing the primer pairs by a scoring system, according to the following criteria:
  - A primer pair is immediately discarded if an in silico non-specific amplicon is returned by PrimerSearch;
  - A score is assigned to each primer pair, according to the cumulative expression level of amplifiable isoforms determined by the transcript quantification process. The distribution of isoform expression is considered and variants expressed more than the quantile threshold determined by the user are assigned a score bonus. For instance, if the threshold is set at 75, that is the 75th percentile quantile, only the top 25% of isoforms with expression levels above this threshold are considered expressed;
  - A primer pair that produces amplicons of approximately 200 base pairs is preferred and receives a higher score, while primer pairs that produce specific amplicons that differ by >25 nucleotides in length are discarded.
    If no splicing junctions are shared by the expressed isoforms, more than one primer pair is returned. For genes with a single isoform, a primer pair is designed considering only the parameters listed in “rock.bakup.”
    The pipeline was designed to take advantage of a CPU’s multithreading capabilities and, although processing a gene at a time, it considers all possible candidate primer pairs designed on all its splicing junctions in parallel.
7. Finally, to exclude other possible non-specific amplifications, primer pairs that satisfy the predefined quality criteria are tested with an in-silico PCR using the reference genome as template and allowing the user-defined percentage of mismatch. Results are saved in the genomic_mismatch.txt file, which should be empty if no amplification with the genome is predicted.

### 2.3 Production and interpretation of the IsoPrimer results

The primary output of the primer design process consists of a primers.xlsx file with three spreadsheets: (i) validation_candidates, which contains a list of the primer pairs that satisfy the predefined quality criteria; (ii) primers_order, which reports primer pairs in a ready-for-order format; (iii) primer_omnibus, which reports all primer pairs designed for all target genes (File 1, available as supplementary data at *Bioinformatics Advances* online).

The output spreadsheets (i) and (iii) report for each primer pair the following information, in separate columns:

- Gene ID;
- IsoPrimer score;
- Transcript ID used as template by Primer3;
- Forward primer sequence;
- Reverse primer sequence;
- The number of expressed isoforms amplified by the primer pair out of the total expressed isoforms. Information about the expressed isoforms is reported in the non-tabular detailed report generated by the pipeline (see below);
- The expression percentage cumulatively accounted for by the amplification with the primer pair; i.e. if a gene has a single expressed isoform amplified by a primer pair, the number is 100 for this primer pair;
- Amplicons predicted by the PrimerSearch in in-silico PCR using the transcriptome sequences reported in the FASTA file provided by the user as a template, represented in the format “name-length,” where name refers to the ID of the transcript sequence targeted by the primer pair and length indicates the length of the amplicon predicted in base pairs.

If the expressed variants of a gene of interest do not share a splicing junction, IsoPrimer returns more than one primer pair. However, all primer pairs must respect the quality criteria defined.

Moreover, for each target gene, a non-tabular detailed report is generated in an outputs folder with the following information (File 2, available as supplementary data at *Bioinformatics Advances* online):

- quantitation of the expression of the isoforms of a gene, supplied by the user or obtained with Kallisto, and the most expressed isoforms (isoforms more expressed than the quantile threshold set by the user);
- isoforms used to design the primer pair and the splicing junction(s) overlapped by primers;
- primer sequences;
- representation of the hybridization of the primers with the matching multialigned isoforms of a gene (ClustalOmega algorithm);
- FASTA sequences of the isoforms matched by the primers designed;
- relevant information returned by Primer3 (e.g. putative secondary structures and hairpins generated within the primers and relative estimates about their stability).

If more than one primer pair is required to amplify all isoforms with an expression level above the quantile threshold chosen by the user, for each target gene, all the aforementioned information is reported for all primer pairs.

### 2.4 Case study: design of primer pairs to validate differentially expressed lncRNAs in the brain of patients with Alzheimer’s disease

IsoPrimer was used to automatically design specific primer pairs for the expressed variants of a set of long non coding RNAs (lncRNAs), that were identified as deregulated in the hippocampal transcriptomic profile of Alzheimer’s disease (AD) patients. The expression trend observed in the RNA-seq data was validated through digital droplet PCR (ddPCR), demonstrating the reliability of IsoPrimer (Filomena *et al.* 2024).

For this aim, we used the following annotation files, downloaded via the following links:

- Genome:
https://ftp.ebi.ac.uk/pub/databases/gencode/Gencode_human/release_33/GRCh38.p13.genome.fa.gz;
- Annotation:
https://ftp.ebi.ac.uk/pub/databases/gencode/Gencode_human/release_33/gencode.v33.annotation.gtf.gz;
- Transcriptome:
https://ftp.ebi.ac.uk/pub/databases/gencode/Gencode_human/release_33/gencode.v33.transcripts.fa.gz.

**Figure 2.**
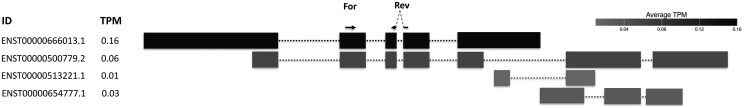
Annealing site of the primer pair designed by IsoPrimer to validate the expression of STARD4-AS1 gene in the case study. The figure reports the four transcripts of STARD4-AS1 gene, annotated in GENCODE v33, together with their expression level, indicated as average of Transcripts per Millions as estimated from RNA-seq data, and the annealing position of the primer pair designed by IsoPrimer. Each transcript is reported with its Ensembl ID. Exons are represented by full boxes; introns are represented by dotted lines. TPM: Transcripts per Millions; For: forward primer; Rev: reverse primer that anneals to an exon-exon junction.

The options that we passed to IsoPrimer via the launcher.sh script are reported in File 3, available as supplementary data at *Bioinformatics Advances* online, the options specified in the Boulder-IO file to design the pairs are reported in File 4, available as supplementary data at *Bioinformatics Advances* online and the output produced by IsoPrimer is reported in File 1, available as supplementary data at *Bioinformatics Advances* online. The annealing site of the primer pair designed by IsoPrimer and chosen for the validation of STARD4-AS1 gene expression is represented in Fig. 2.

## 3 Discussion

To the best of our knowledge, IsoPrimer is the only tool able to guide researchers in the design of PCR primers for validating RNA-seq data, ensuring an accurate gene expression quantification by targeting all the expressed isoforms of a gene of interest, as indicated by RNA-seq data, generated in-house or obtained from public repositories. IsoPrimer runs locally and optimizes primer pair design based on the expression levels of splicing variants of the gene of interest, offering the possibility of tweaking the parameters of the pipeline components, according to the specific experimental needs. The pipeline returns the best possible candidate primer pairs according to the parameters chosen. IsoPrimer offers flexibility by allowing users to design primer pairs according to the expression values reported in a transcript expression table generated by the users with quantification tools of their choice not included in IsoPrimer (formatted as the table shown in Table 1, available as supplementary data at *Bioinformatics Advances* online) or enabling transcript quantitation using the default Kallisto tool, integrated within IsoPrimer. Alternatively, the user can also manually populate the transcript quantification table with arbitrary expression values to prioritize specific sets of isoforms. IsoPrimer considers all possible primer pairs that satisfy the requirements both in terms of sequence features, via the Primer3 parameters, and in terms of specificity, by researching putative non-specific amplifications both in the genome and in the transcriptome. The individual programs used to build IsoPrimer are widely used and well documented, and the robustness of the computational approach of IsoPrimer was demonstrated by the validation by ddPCR of the deregulated expression of a set of lncRNAs resulted differentially expressed in the hippocampal transcriptomic profile of Alzheimer’s disease (AD) patients (Filomena *et al.* 2024).

A limitation of IsoPrimer may lie in the reliance on current annotation since the success of a primer design run depends on the reliable annotation of the splicing isoforms of a gene of interest. However, the dependence on annotation is an issue that is common to all reference-based bioinformatic approaches.

In conclusion, we believe that IsoPrimer can represent a valuable support to perform comprehensive gene expression quantification by qPCR, especially in case of complex gene splicing patterns, as it enables the design of PCR primers that specifically target all expressed isoforms of a gene. It is an end-to-end pipeline, from primer design and in silico specificity assessment through to final primer prioritization, thereby enhancing the accuracy and comprehensiveness of downstream expression quantification.

## Supplementary Material

vbaf171_Supplementary_Data

## Data Availability

The IsoPrimer pipeline presented in this article is available in GitHub alongside its README file at https://github.com/BioinfoUNIBA/IsoPrimer.
