# Supplementary material for: IsoPrimer: a pipeline for designing isoform-aware primer pairs for comprehensive gene expression quantification
Source: Bioinform Adv. 2025 Jul 15;5(1):vbaf171. doi: 10.1093/bioadv/vbaf171 (PMC12311343; doi:10.1093/bioadv/vbaf171)
Supplement: vbaf171_Supplementary_Data [file vbaf171_supplementary_data.zip › Supplementary_Table_1_revision.docx]

**Supplementary Table 1.** **example of a customised Kallisto quantification table to design primers for the manually picked variant Odc1-207 (ENSMUST00000222250).** High expression (e.g., 1e+06) is assigned to the isoform of interest, prompting IsoPrimer to prioritize it for primer design.

| t_name | tseq |
| --- | --- |
| ENSMUST00000171737 | 0 |
| ENSMUST00000222617 | 0 |
| ENSMUST00000221701 | 0 |
| ENSMUST00000220947 | 0 |
| ENSMUST00000221613 | 0 |
| ENSMUST00000220849 | 0 |
| ENSMUST00000221354 | 0 |
| ENSMUST00000222250 | 1e+06 |
