# Supplementary figures and images for: IsoPrimer: a pipeline for designing isoform-aware primer pairs for comprehensive gene expression quantification
Source: Bioinform Adv. 2025 Jul 15;5(1):vbaf171. doi: 10.1093/bioadv/vbaf171 (PMC12311343; doi:10.1093/bioadv/vbaf171)

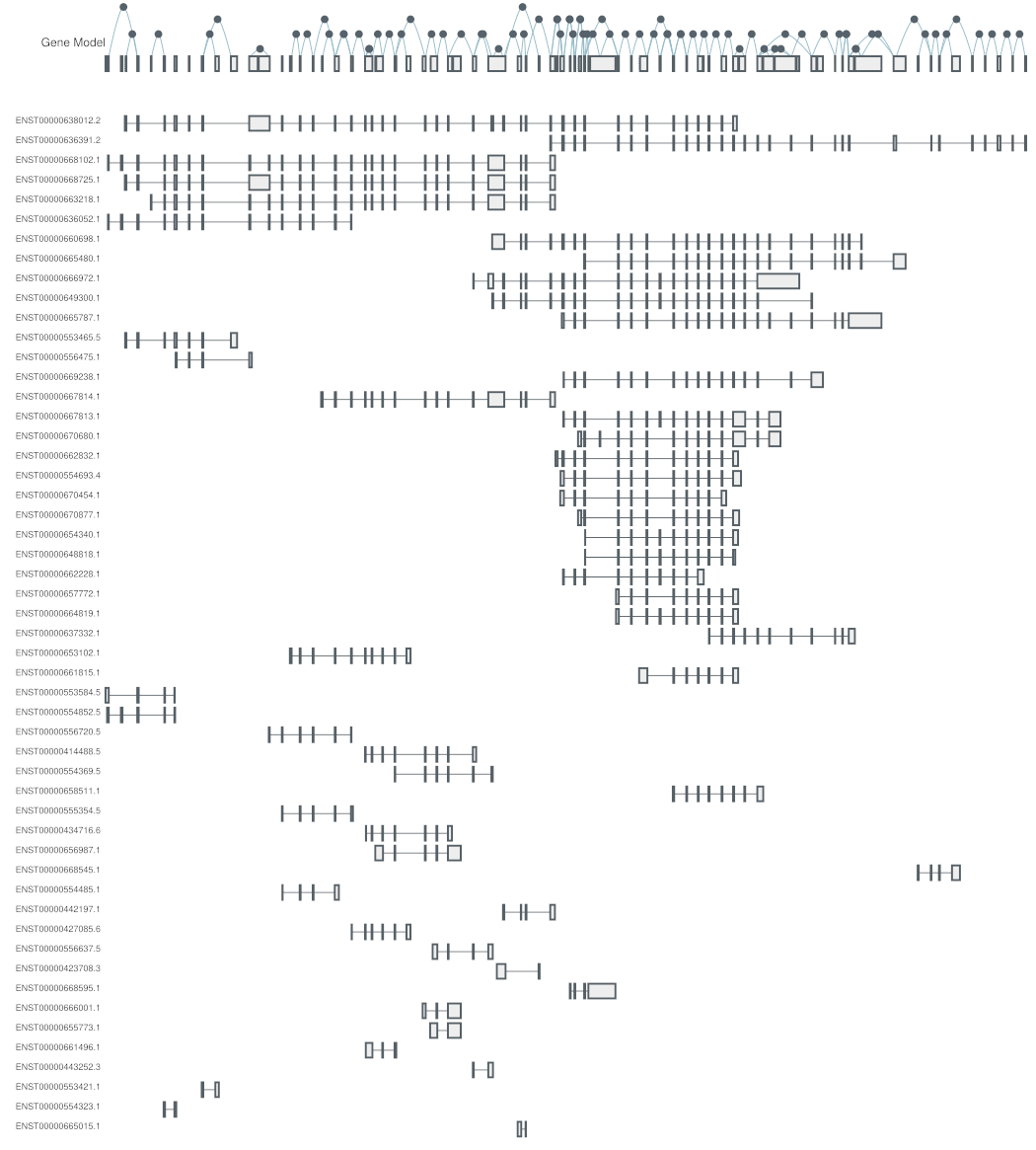

Supplement: vbaf171_Supplementary_Data [file vbaf171_supplementary_data.zip › Supplementary_Figure_1.jpeg]
